# Large Extent of Disorder in Adenomatous Polyposis Coli Offers a Strategy to Guard Wnt Signalling against Point Mutations

**DOI:** 10.1371/journal.pone.0077257

**Published:** 2013-10-09

**Authors:** David P. Minde, Martina Radli, Federico Forneris, Madelon M. Maurice, Stefan G. D. Rüdiger

**Affiliations:** 1 Cellular Protein Chemistry, Bijvoet Center for Biomolecular Research, Utrecht University, Utrecht, The Netherlands; 2 Crystal and Structural Chemistry, Bijvoet Center for Biomolecular Research, Utrecht University, Utrecht, The Netherlands; 3 Department of Cell Biology, University Medical Center Utrecht (UMCU), Utrecht, The Netherlands; Monash University, Australia

## Abstract

Mutations in the central region of the signalling hub Adenomatous Polyposis Coli (APC) cause colorectal tumourigenesis. The structure of this region remained unknown. Here, we characterise the Mutation Cluster Region in APC (APC-MCR) as intrinsically disordered and propose a model how this structural feature may contribute to regulation of Wnt signalling by phosphorylation. APC-MCR was susceptible to proteolysis, lacked α-helical secondary structure and did not display thermal unfolding transition. It displayed an extended conformation in size exclusion chromatography and was accessible for phosphorylation by CK1ε *in vitro*. The length of disordered regions in APC increases with species complexity, from *C. elegans* to *H. sapiens*. We speculate that the large disordered region harbouring phosphorylation sites could be a successful strategy to stabilise tight regulation of Wnt signalling against single missense mutations.

## Introduction

Adenomatous Polyposis Coli (APC) is mutated in around 80% of somatic colorectal cancer patients [1]. APC is one of the central hubs involved in the spatiotemporal regulation of key Wnt signalling components [2,3]. It interacts with the Wnt signalling scaffolds WTX and Axin, which locally concentrate the kinases GSK3β and CK1 within the destruction complex for tightly controlled degradation of the proto-oncoprotein β-catenin [4-16]. APC binding to β-catenin and Axin is essential for effective downregulation of β-catenin and tumour suppression [17]. Eleven β-catenin binding repeats are scattered over a region extending from residue L1021 to D2059, interspersed by 3 Axin binding repeats [18]. Seven β-catenin binding repeats are tunable in affinity by phosphorylation and are referred to as 20aa repeats, while four 15aa repeats remain nonphosphorylated [12,19-21]. 

The structural understanding of APC function is limited. APC contains three α-helical domains in the N-terminus: a coiled coil (APC-A2-I55) mediates homodimerisation, APC- G126-H250 binds to Crm1 and APC-R303-N775 constitute an armadillo fold that binds to the phosphatase PP2A [22-26]. Remarkably, globular domains are unknown in the 2000 amino acid large C-terminal region of APC. We previously predicted those 2000 amino acids as largely intrinsically disordered [18]. Interestingly, this segment contains the most frequently mutated stretch of APC, the mutation cluster region (MCR), which contains three β-catenin binding 20aa repeats [27,28]. It is unclear how the structural properties of APC-MCR relate to β-catenin recognition and regulation by phosphorylation.

Here we characterised structural properties of APC-MCR. We show that APC-MCR is susceptible to proteolysis, lacks both α-helical secondary structure and thermal unfolding transitions. The protein displayed extended shape and was accessible for phosphorylation. Based on those data, we propose a mechanism for how large intrinsically disordered regions in APC and other Wnt signalling scaffolds benefit robustness of the Wnt signalling cascade.

## Results

### Charged residues cluster in distinct regions of APC-MCR

**Figure 1:**
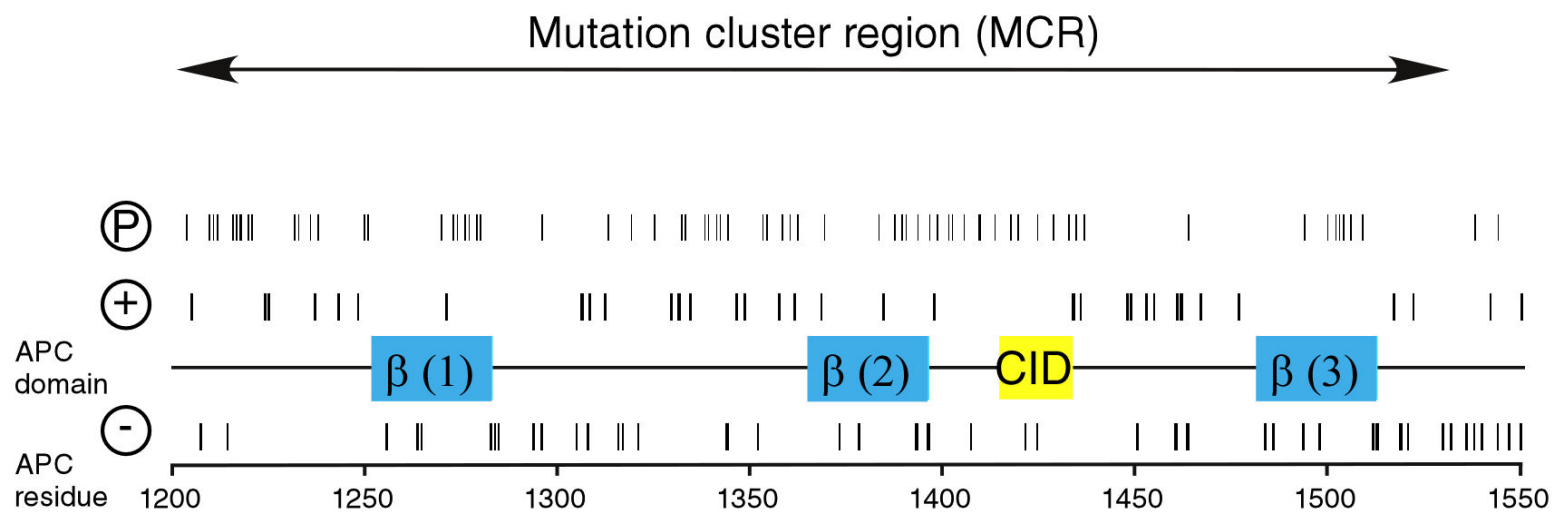
Charge distribution of APC-MCR. Charges present in the primary structure, putative phosphorylation sites and regulatory important motifs are indicated. A large number of putative phosphorylation sites scatter over the entire MCR (Phosida predictor for S/T phosphorylation). Two of the three 20 amino acid repeats (blue), bind to β-catenin (β). A short peptide stretch called “CID” (yellow) was recently implicated in Wnt/β-catenin downregulation [4,70].

When setting out to characterise the structural features of APC-MCR (APC-S1202-K1551), we noted that folded domains are unknown in the C-terminal 2000 amino acids of APC. Bioinformatic studies predicted a large extent of disorder for this region [18,29]. We, therefore, first analysed the distribution of charges, which is a key determinant for appearance of disordered regions in proteins [30,31] (Figure 1). Surprisingly, we did not observe a strong enrichment of charges in the putatively disordered APC-MCR region: Both positive charges and negative charges (9.7% and 12.2%) were close to the corresponding averages in the UniProtKB/Swiss-Prot data base (11.1% and 12.3%).

### APC-MCR is susceptible to proteolysis

**Figure 2:**
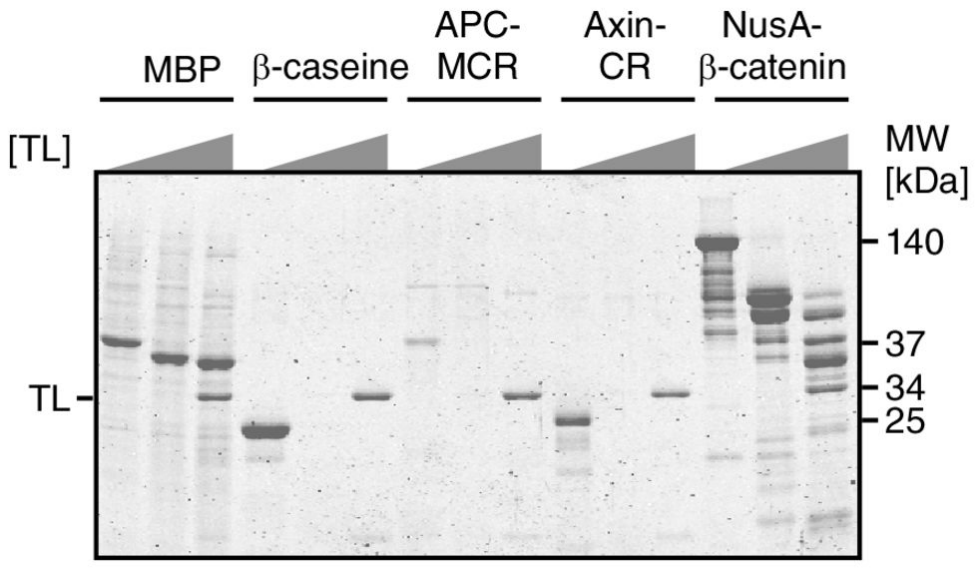
MCR is susceptible to TL digestion. Resistance of well-characterised folded and unfolded proteins is compared with MCR by using TL concentrations of 0 g/L, 0.001 g/L and 0.1 g/L. Folded protein MBP (diamond), is resistant against the highest protease concentration while β-caseine and Axin CR are already cleaved at low protease concentration (0.001g/L). A fusion construct of NusA and β-catenin gives several high molecular weight bands, likely due to the presence of at least one large protease susceptible internal linker segment [65].

As accessibility of hydrophobic amino acids is crucial for protein interactions and structure formation, we went on to probe the accessibility of the hydrophobic residues in APC-MCR by titrating the protease Thermolysin (TL), which preferentially cleaves near exposed hydrophobic amino acids in unfolded protein regions [32-36]. To estimate the degree of globular structure in APC-MCR, we compared the extent of cleavage at 4°C for APC-MCR along with controls that are known to be fully folded (MBP), fully unfolded (Axin-CR, β-caseine) or partly folded (NusA-β-catenin) [37]. For each of these proteins, we compared the remaining protein after incubation at 4°C in the absence or in the presence of 0.001 g/L TL or 0.1 g/L TL. The folded core of MBP was resistant against proteolysis even at the highest TL concentration (Figure 2). The two intrinsically disordered proteins (IDPs) AxinCR and β-caseine, however, were entirely digested at low TL concentration (0.001 g/L). Likewise, the band of APC-MCR disappeared at this TL concentration. We conclude that protease susceptibility of APC-MCR is similar to that of the IDPs Axin-CR and β-caseine.

### APC-MCR lacks secondary structure in non-denaturing buffer

**Figure 3:**
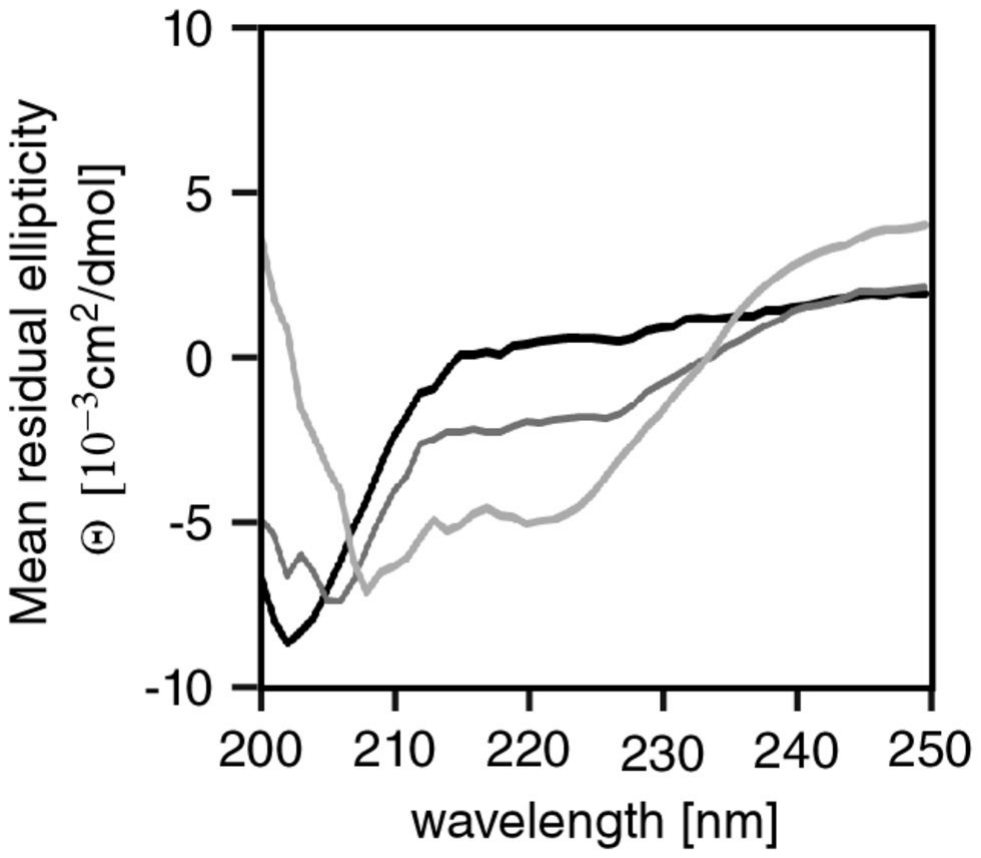
MCR lacks secondary structure. Far-UV CD spectra of APC-MCR in the absence (black) and presence of TFE (dark grey, 20%; light grey, 80%) in 10 mM Na-phosphate buffer (pH 7.2), 50 mM NaF, and 0.5 mM TCEP.

We now set out to analyse secondary structure of APC-MCR by circular dichroism (CD) spectroscopy. We varied the content of TFE to investigate if α-helical structure can be induced by this co-solvent, which is frequently used to induce or stabilise α-helical secondary structure [38-40]. In non-denaturing buffer and absence of TFE, we observed a minimum near 200 nm and absence of a negative peak at 220 nm indicating low α-helical structure content (Figure 3) [41]. With addition of TFE to 20 or 80 %, increasing content of α-helix was induced. At 80% TFE, a concentration known to stabilise α-helical structure in peptides and proteins, APC-MCR showed a predominantly α-helical spectrum with negative peaks at 208 and 220 nm [39]. Compared to the intrinsically disordered Axin-CR, APC-MCR has a lower content of α-helicity at TFE concentrations up to 20% [37]. We conclude that APC-MCR is devoid of α-helical secondary structure in non-denaturing buffer, although TFE can induce α-helicity.

### APC-MCR does not fold or unfold upon heating

**Figure 4:**
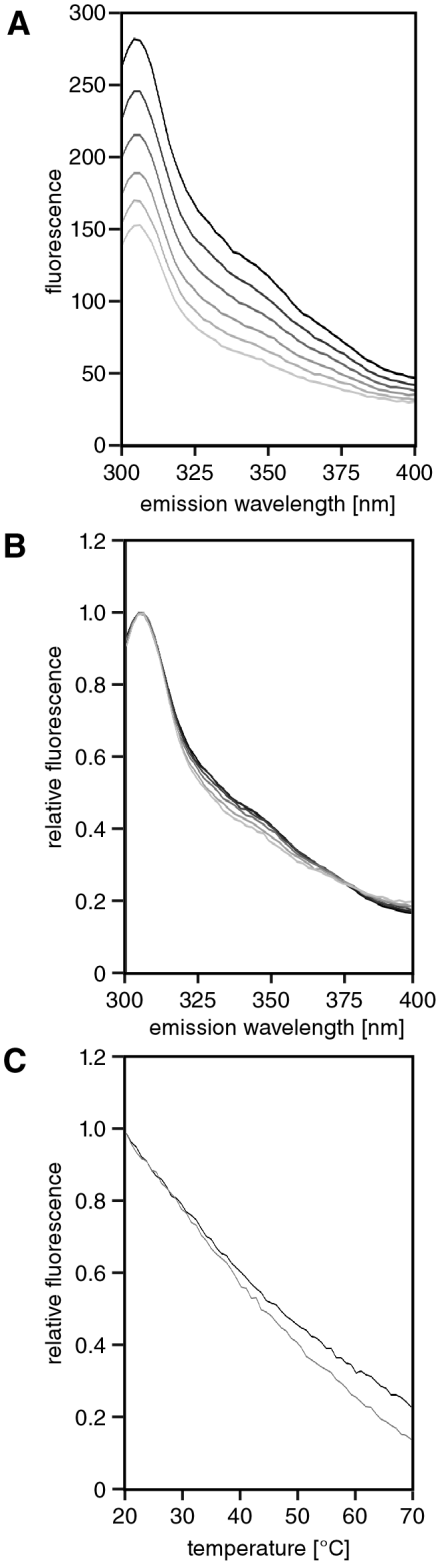
APC-MCR lacks a cooperative unfolding transition. **A**, Intrinsic protein fluorescence spectra of APC-MCR measured in 1°C steps from 20°C to 70°C, indicated by a greyscale gradient from black to light grey**. B**, Comparison of intrinsic tyrosine fluorescence spectra normalised to the maxima. Experiment and colour code as in A. C Temperature dependence of intrinsic tyrosine fluorescence emission of MCR upon stepwise increase of temperature at 340 nm (black) and 304 nm (grey) normalised on maxima of emission.

To investigate tertiary structure of APC-MCR, we monitored the temperature dependence of its intrinsic protein fluorescence. We heated the APC-MCR sample in steps of 1°C from 20°C to 70°C and acquired fluorescence emission spectra at each temperature. We observed a linear decrease of fluorescence emission with increasing temperature, as it is typical for protein in the absence of global conformational changes (Figure 4A,B,C) [42]. We neither detected any thermally induced transition nor temperature-dependent changes in the spectral shape (Figure 4A, B). We conclude that temperature increase does not induce a structural transition that changes the environment of the intrinsic fluorescence probes in APC-MCR.

### APC-MCR samples an extended conformational ensemble

**Figure 5:**
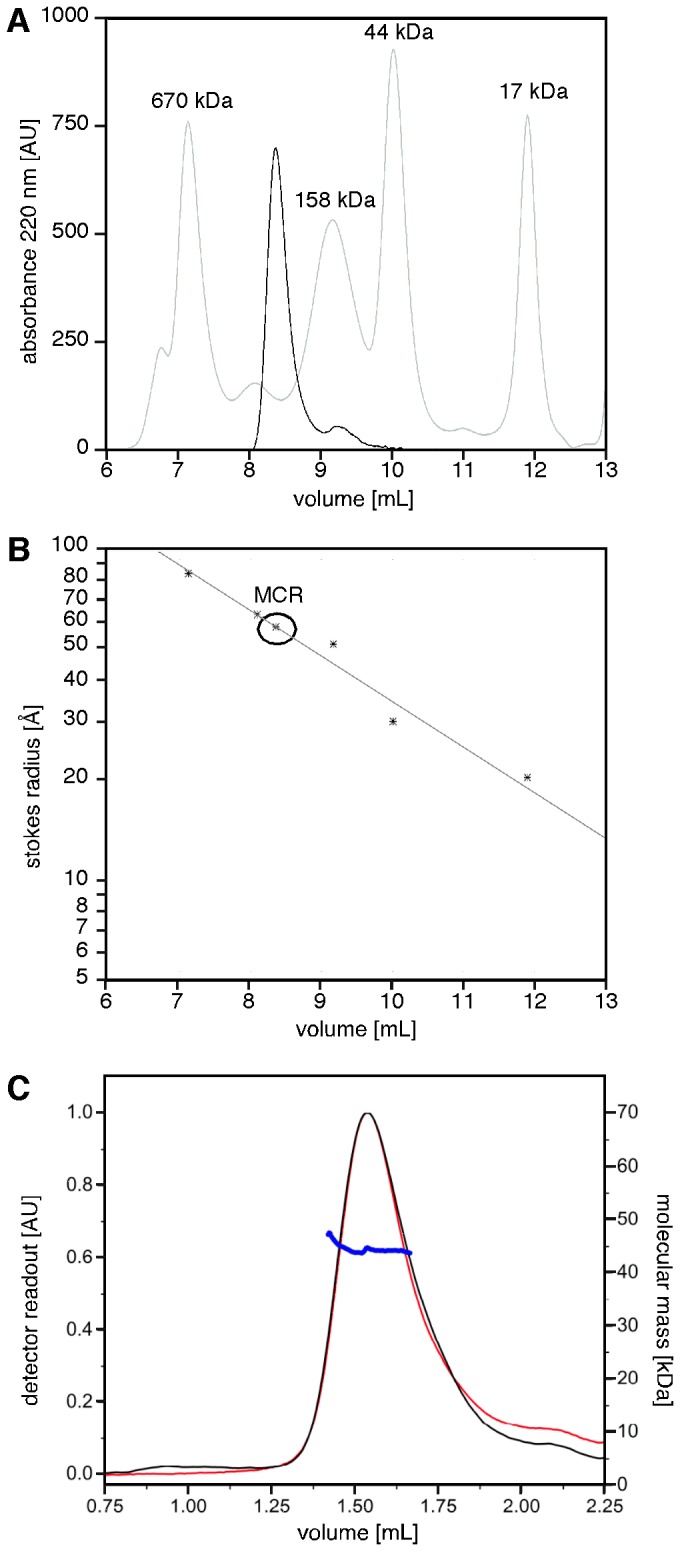
APC-MCR runs at a higher apparent molecular weight upon size exclusion chromatography. **A**, APC-MCR was applied to analytical size exclusion chromatography. MCR eluted after 8.36 min between the indicated globular size standards. By semilogarithmic fitting to the molecular weights of the standards, an apparent molecular weight of 220 kDa has been determined for APC-MCR. **B**, The straight line indicates a semilogarithmic fit to the known stokes radii of the indicated standard proteins. According to this analysis, MCR has a stokes radius of 5.9 nm. **C**, SEC-MALLS reveals that the ySUMO-APC-MCR fusion protein is monomeric. ySUMO-APC-MCR eluted as a single peak with a mass of 44.4 ± 4.0 kDa (UV absorption at 220 nm, black, arbitrary units; differential refractive index, red, arbitrary units; Molecular mass, blue, kDa).

Next, we analysed the global shape of APC-MCR by Size-Exclusion Chromatography (SEC). For size comparison, we calibrated the column with an established range of globular proteins of known mass from 17 kDa (myoglobin) to 670 kDa (thyroglobulin). The elution time of APC-MCR corresponded to an apparent molecular weight of 200 kDa, 5-fold larger than expected based on its molecular weight of 40 kDa (Figure 5A,B). We conclude that APC-MCR is either predominantly extended or oligomeric. To determine the oligomeric propensity of APC-MCR, we determined the molecular weight of the ySUMO-APC-MCR fusion protein by Size Exclusion Chromatography – Multi-Angle Laser Light Scattering (SEC-MALLS) (Figure 5C). The protein eluted as a single monomeric peak with a molecular mass of 44.4 ± 4.0 kDa. We conclude that APC-MCR is an extended intrinsically disordered region.

### APC-MCR is accessible for phosphorylation by CK1ε

The extended nature of APC-MCR may facilitate posttranslational modifications such as phosphorylation. We predicted potential phosphorylation sites using algorithm PHOSIDA, identifying 69 possible sites [43]. Phosphorylation of just eight (12%) of the 69 predicted sites in APC-MCR would already revert the net charge of APC-MCR (Figure 1). We, therefore, investigated whether the Wnt pathway kinase CK1ε would phosphorylate APC-MCR, as it would be expected if the structure was extended. 

**Figure 6:**
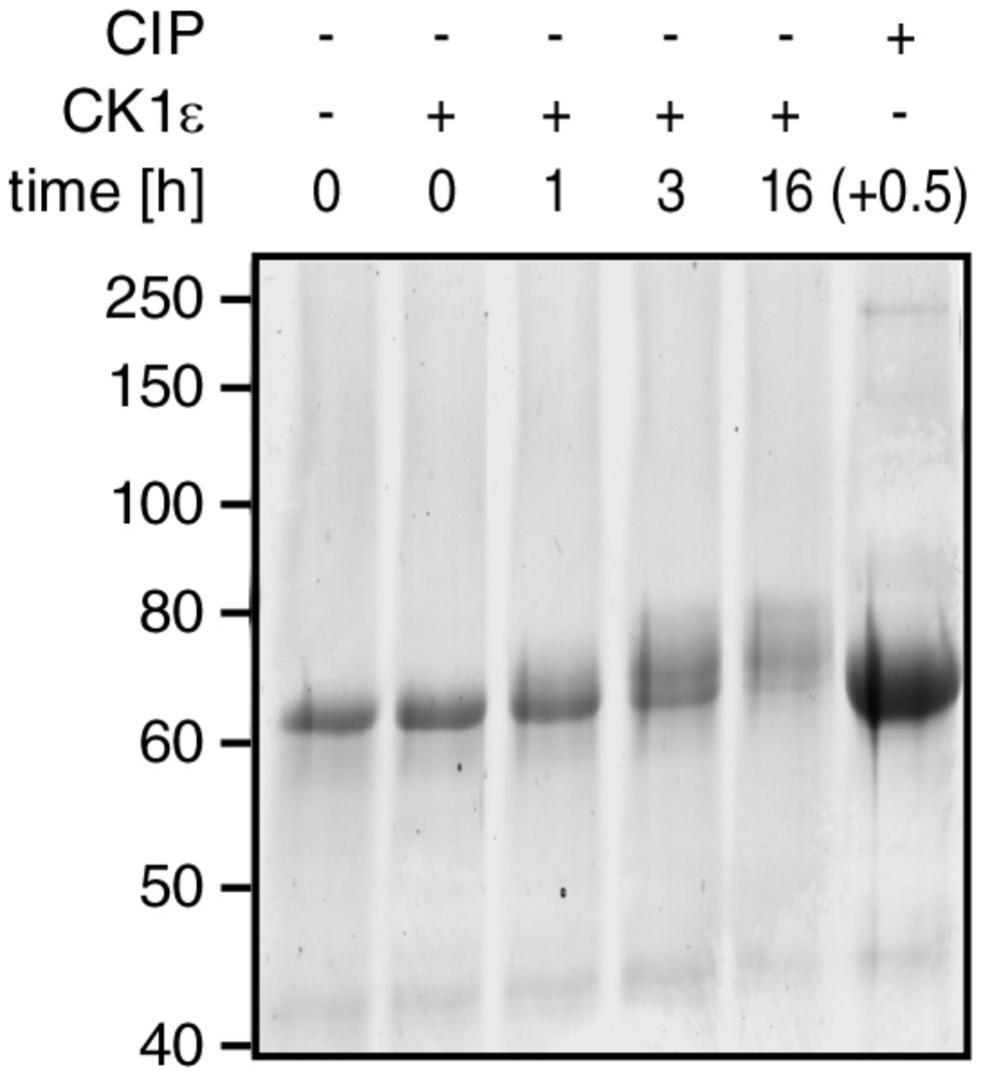
MCR can be phosphorylated by CK1ε. ySUMO-MCR was incubated for the times indicated with CK1ε. The bandshift of phosphorylated ySUMO-MCR after 16 h phosphorylation was fully reversed by heat inactivation of CK1ε followed by dephosphorylation with CIP for 30 min (last lane).

In the presence of ATP and CK1ε, we incubated APC-MCR for up to 16 h and subsequently probed for an eventual change in migration pattern on SDS-PAGE. Additional negative charges locally prevent binding of SDS and, therefore, reduce mobility of the phosphorylated protein in the gel [21,44-47]. We observed a significant decrease in electrophoretic mobility of phosphorylated APC-MCR already after 1 h and a more pronounced shift upon further incubation, as it would be expected upon addition of additional negative charge (Figure 6). After 16 h exposure to the kinase, the unphosphorylated APC-MCR band disappeared, indicating that no unphosphorylated APC-MCR was left. This effect on electrophoretic mobility was reversed upon subsequent addition of a phosphatase to phosphorylated samples after thermal inactivation of CK1ε Figure 6). Our results demonstrate that APC-MCR can be phosphorylated by CK1ε. Our finding are consistent with previous reports that a short peptide of the third 20aa repeat of APC (APC-D1498-F1517) can indeed be phosphorylated on up to six sites, one more than predicted by PHOSIDA [21].

### The Wnt signalling scaffolds WTX, APC, Axin contain large disordered regions

**Figure 7:**
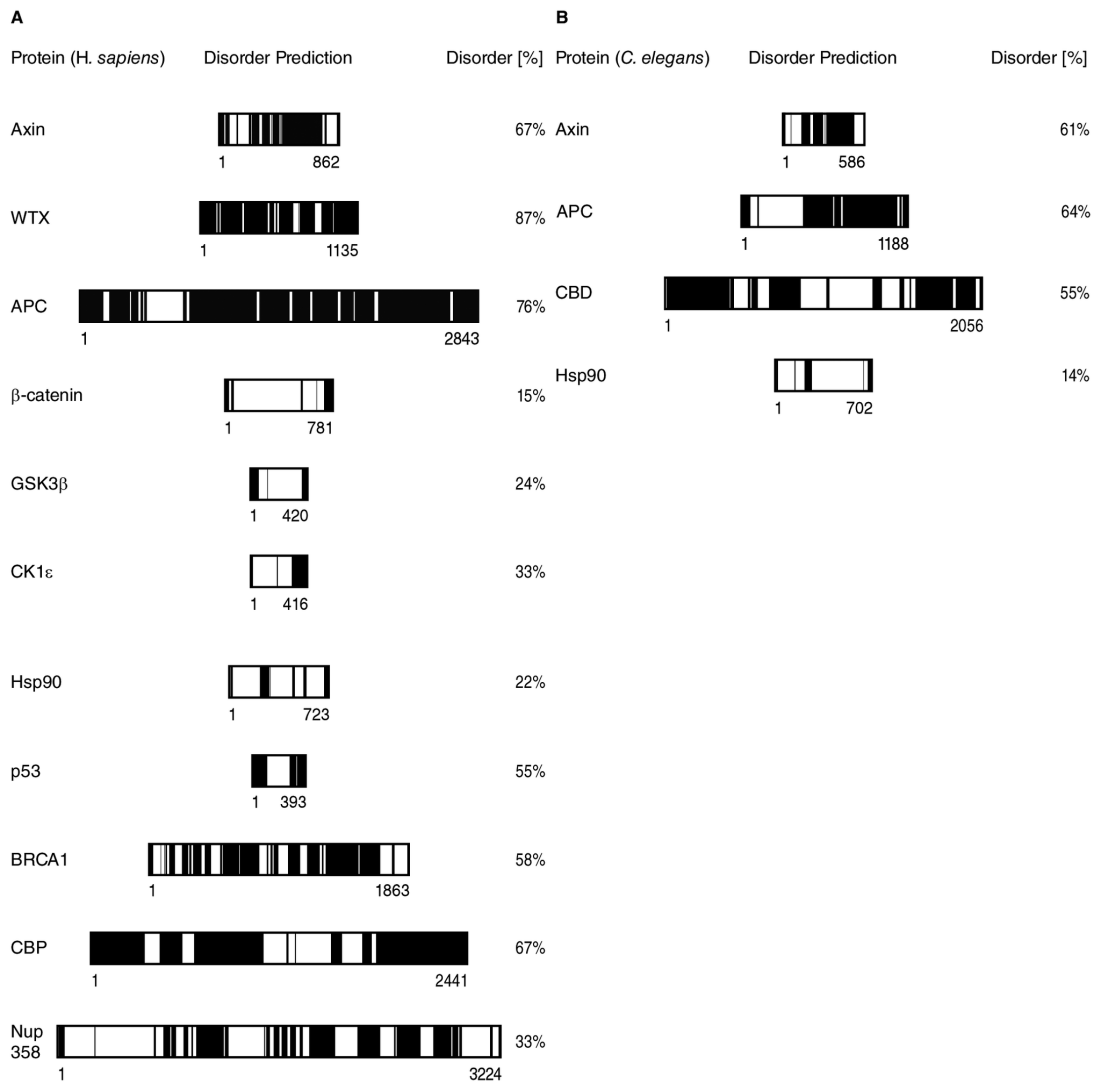
The three Wnt signalling hubs APC, Axin, WTX contain large intrinsically disordered regions. **A**, Meta-predictions of disorder using the PONDR-FIT algorithm are displayed. (black for scores >= 0.5 and white for scores < 0.5). **B**, Same as A, for *C. elegans* homologues of APC, Axin, Hsp90, CBP.

We wondered whether the high degree of intrinsic disorder is unique to APC-MCR. Therefore, we compared the disorder tendency of APC-MCR to that of other members of the Wnt signalling cascade (WTX, APC, Axin, β-catenin, GSK3β, CK1ε) as well as unrelated hubs and scaffold proteins (Hsp90, p53, BRCA1, CREB-binding Protein (CBP), Nup358) using the metaprediction algorithm PONDR-FIT [48-56]. We confirmed the presence of a large intrinsically disordered central region in Axin and large parts of APC (Figure 7A) [10,37,57]. Interestingly, also the third scaffold of the Wnt destruction complex, WTX, was predicted to exhibit an unusual high degree of disorder (87%; Figure 7A). Indeed, we noticed typical features of an intrinsically disordered protein in WTX: low sequence complexity, two-fold enriched content of the disorder promoting residues Glu, Pro, Ser, accounting for 31.1 % of all residues (compared to 15.2% in the average of UniProt), and several repeats of single amino acids, e.g. in patches of (Glu)_7_, (Asp)_4_, (Pro)_3_ and (Ser)_4_ [58]. In comparison to other Wnt signalling proteins and several hubs or scaffolds, we noticed that the Wnt signalling scaffolds WTX (87%), APC (76%) and Axin (67%) exhibit a higher degree of disorder than most of the control proteins (average 38% (similar to the predicted fraction of disorder in most multicellular proteomes of around 40 %); only CBP shows a similar extent of disorder (67%) [59]. We conclude that in agreement with previous bioinformatic and experimental work, disorder is a key feature in scaffolding complex protein interaction networks [60,61].

We now wondered how the fraction, distribution and absolute amount of disordered residues in Axin and APC vary among evolutionary distant orthologues from species with low (*C. elegans*) to high (*H.sapiens*) biological complexity. Our comparison of disorder predictions showed for five orthologues of APC and Axin a significant increase in disorder along with species complexity (Figure 7B, C). For APC, the total number of predicted disordered residues increased from 761 in *C. elegans* to 2165 in *H. sapiens*, while the relative disorder content remained considerably higher than the (bioinformatically characterised) eukaryotic proteome average of around 40% (64% in *C. elegans* and 76% in *H. sapiens*) [59]. This increase in disorder tendency is remarkable because random mutations would have been expected to reduce both the fraction and absolute length of disordered regions during evolution [62,63] This implies that extended disordered regions in APC convey a selective advantage for complex organisms.

## Discussion

Our results established that APC-MCR is extended and intrinsically disordered. High susceptibility to cleavage by TL, absence of α-helicity, lack of intrinsic fluorescence temperature transitions and aberrantly fast SEC elution support this conclusion. Our data are largely consistent with previous studies on short APC-MCR peptides of all isolated β-catenin binding repeats and a larger fragment (APC-S1362-K1745) downstream of APC-MCR [12,19,20,64,65]. A 21 kDa APC fragment containing the second and third 20aa repeats (APC-S1340-E1536) has aberrant mobility in SEC, resulting in an apparent weight of 70 kDa, which supports our findings [64]. Similarly, mixing this 21 kDa fragment with an 83 kDa β-catenin-GST (apparent weight on SEC 120 kDa), yields a complex of 190 kDa apparent weight [64]. This is consistent with assuming extended structure of APC-S1340-E1536, similar to our findings for APC-S1202-K1551.

Our findings that APC-MCR lacks secondary and tertiary structure are also consistent with crystallographic studies of APC fragments (APC-S1362-E1540 and APC-D1484-D1498), in which regions corresponding to individual 20aa repeats lack unique globular structure and bind to β-catenin in an extended conformation [12,19]. Also, NMR and CD data revealed that the APC-S1340-E1536 spanning the 20aa repeats two to four lacks stable secondary and tertiary structure [20]. We conclude that, based on a diverse set of complementary experimental studies, APC-MCR contains large intrinsically disordered regions. Disordered scaffolds have been recently proposed to act as stochastic machines [66]. Our study provides evidence that APC has all features required for a stochastic machine.

Notably, not only the Wnt scaffolds APC and Axin are to a large extent intrinsically disordered, our bioinformatic analysis suggested also for WTX a high tendency to disorder (Figures 7) [18,29,37]. Intrinsic disorder, we thus conclude, is a recurring theme in large regions of three scaffolds in Wnt signalling, which might facilitate binding to ordered regions in other Wnt pathway interactors. Common to all three scaffolds is that they offer redundant binding sites for several folded proteins. The connection of those sites with disordered sequences transforms intermolecular reactions into entropically more effective intramolecular reactions, largely independent of which binding sites are used. 

A particular interesting question is how the disordered nature of APC-MCR corresponds to colorectal tumourigenesis. Only few cases are known where a single missense mutation in APC’s disordered regions has drastic clinical consequences [18]. This is in marked contrast to point mutations in folded domains such as the p53 core domain or in the relatively short disordered N-terminal region of β-catenin [51]. We speculate that the large extent of disorder in APC regions that host numerous β-catenin and Axin binding repeats may increase robustness against single missense mutations [18,29,67]. Due to the high redundancy of β-catenin binding motifs in APC, a reduced function of a single repeat is likely to be tolerated in most cases, in particular when connected with disordered, dynamic linker segments. This is consistent with a relatively low prevalence of missense mutant APC in colorectal cancer patients [18]. The combination of a redundant set of binding sites for interactors and extended intrinsic disorder and presence of multiple potential phosphorylation sites might be crucial to protect Wnt signalling fidelity against mutational impact.

## Methods

### Protein Purification

All used protein production constructs were prepared from human cDNA. The β-catenin was recloned in a modified pET50b (Novagen) encoding a TEV cleavage site before the start of the β-catenin sequence. APC-MCR (S1202-K1551) was recloned as a Ulp1-cleavable ySUMO fusion. Axin-CR was prepared as previously described [37]. CK1ε was prepared as described [68]. The proteins were produced in *E. coli* BL21(DE3)Rosetta2* (a strain obtained by cotransformation of the protein production constructs with the pRARE2 plasmid from Novagen into the BL21(DE3)* strain from Invitrogen). Upon production at 15°C, we purified the proteins using Poros20MC resin for metal affinity purification. Subsequently, tags were cleaved on ice using TEV or Ulp1 protease using conditions described earlier [37]. The APC-MCR protein was further purified by Poros20HQ anion exchange chromatography for tag removal and polishing. For the SEC-MALLS experiments, ySUMO-APC-MCR was purified by metal-chelate affinity chromatography (Poros 20MC) and anion exchange chromatography (Poros 20HQ and MonoQ (GE Healthcare)) and concentrated to 50 µM using a Vivaspin 20 concentrator (10,000 MWCO PES, Sartorius Stedim Biotech) in (20 mM Tris pH 8, 200 mM KCl, 0.8 mM TCEP).

### TL titration

TL was prepared as described [35]. Stock solutions of 5 g/L were diluted to 0.1 g/L or 0.001 g/L. All reactions were incubated on ice for 30 min in a buffer containing Hepes 20 mM pH 7.2, 300 mM KCl, 10 mM CaCl_2_ and 5 mM DTT as reducing agent for the cytosolic proteins Axin-CR, APC-MCR, β-catenin. Subsequent quenching of the reaction was achieved by addition of a Laemmli sample buffer supplemented with 50 mM EDTA. All samples were analysed by SDS-PAGE and subsequent fluorescence-enhanced detection in an Odyssey scanner (LiCor) [69].

### Fluorescence Analysis

Purified APC-MCR (30 μM) in 10 mM sodium phosphate (pH 7.2), 150 mM NaCl, and 1 mM DTT was measured using a fluorescence spectrophotometer (Perkin-Elmer LS55) with a 1.5-ml cuvette (Hellma) with a magnetic stirrer using the software Fluo_pe (D. Veprintsev; Paul Scherrer Institute, CH). Fluorometric analysis was performed at increasing temperatures ranging from 20°C to 70°C for APC-MCR, with a 1°C step increase. Fluorescence emission was measured from 300 to 400 nm with excitation at 280 nm.

### CD spectroscopy

CD spectra were recorded with a J-810 spectropolarimeter (Jasco). Purified MCR (10 μM) in 10 mM Na-phosphate (pH 7.2), 50 mM NaF, and 0.5 mM TCEP was applied on a 1mm Quartz cell (Hellma). Far-UV spectra were collected over a range of 190–260 nm using Jasco software at a scanning speed of 50 nm/min and at a data pitch of 1 nm, averaged over 10 acquisitions. Spectra were corrected for buffer contributions by substracting buffer reference spectra. Different concentrations of TFE were used: 0%, 20% and 80% (vol/vol).

### Size Exclusion Chromatography

Analytical gel filtration of purified MCR at a concentration of 50 μM was performed on an Äkta Purifier (GE Healthcare) using a Bio-Silect SEC 250 analytical column (300 mm×7.8 mm) with a guard column (50 mm×7.8 mm; Bio-Rad) equilibrated with 25 mM Tris–HCl (pH 7.2), 150 mM NaCl, and 1 mM TCEP. MCR was eluted at a flow rate of 1 ml/min, and the elution profile was recorded by continuously monitoring UV absorbance at 280 nm and 220 nm. The calibration curve was obtained using standards of known size and molecular mass from a lyophilized protein test mix (Bio-Rad), including thyroglobulin (670 kDa), γ globulin (158 kDa), ovalbumin (44 kDa), myoglobin (17 kDa), and vitamin B12 (1.3 kDa).

### Size Exclusion Chromatography – Multi-Angle Laser Light Scattering (SEC-MALLS)

SEC-MALLS had been used to determine the oligomerisation propensity of APC-MCR using the ySUMO-APC-MCR fusion protein. For each SEC-MALLS run, 10 µl of 50 µM ySUMO-APC-MCR had been injected into a Superdex 200 5/150 GL gel filtration column (GE Healthcare) and separated with a flow rate of 0.2 ml/min in 20 mM Tris pH 8, 200 mM KCl, 0.8 mM TCEP. For molecular weight characterisation, light scattering was measured with a miniDAWN TREOS multi-angle light scattering detector (Wyatt), connected to a differential refractive index monitor (Shimadzu, RID-10A) for quantitation of the protein amount. Chromatograms were collected, analysed and processed by ASTRA6 software (Wyatt, using an estimated dn/dc value of 0.185 ml/g). The calibration of the instrument was verified by injection of 10 µl of 3 g/l monomeric BSA (Sigma-Aldrich).

### Phosphorylation assay

ySUMO-APC-MCR was treated with 10 nM CK1ε and 100 μM ATP for up to 16 h. For a dephosphorylation control, we heat-inactivated the CK1ε after 16 h phosphorylation and used Calf-intestine Phosphatase (CIP, NEB) according to the protocol of the manufacturer. 
